# State Observer Design for Delayed Genetic Regulatory Networks

**DOI:** 10.1155/2014/761562

**Published:** 2014-05-22

**Authors:** Li-Ping Tian, Zhi-Jun Wang, Amin Mohammadbagheri, Fang-Xiang Wu

**Affiliations:** ^1^School of Information, Beijing Wuzi University, Beijing 101149, China; ^2^College of Mathematics and Statistics, Hebei University of Economics and Business, Shijiazhuang, Hebei 050061, China; ^3^Division of Biomedical Engineering, University of Saskatchewan, Saskatoon, SK, Canada S7N 5A9; ^4^Department of Mechanical Engineering, University of Saskatchewan, Saskatoon, SK, Canada S7N 5A9

## Abstract

Genetic regulatory networks are dynamic systems which describe the interactions among gene products (mRNAs and proteins). The internal states of a genetic regulatory network consist of the concentrations of mRNA and proteins involved in it, which are very helpful in understanding its dynamic behaviors. However, because of some limitations such as experiment techniques, not all internal states of genetic regulatory network can be effectively measured. Therefore it becomes an important issue to estimate the unmeasured states via the available measurements. In this study, we design a state observer to estimate the states of genetic regulatory networks with time delays from available measurements. Furthermore, based on linear matrix inequality (LMI) approach, a criterion is established to guarantee that the dynamic of estimation error is globally asymptotically stable. A gene repressillatory network is employed to illustrate the effectiveness of our design approach.

## 1. Introduction

Recently nonlinear differential equations have been proposed to model genetic regulatory networks. Based on this model, stability of genetic regulatory networks has been intensively studied, which is believed useful in designing and controlling genetic regulatory networks. In [1], sufficient and necessary local delay-independent stability conditions are given for several types of simplified genetic regulatory networks with a single time delay. In [2, 3], we present some sufficient and necessary conditions of local delay-independent stability conditions for general genetic regulatory networks with a single time delay and multiple time delays. Some sufficient conditions for global stability of genetic regulatory networks have been derived based on LMI approaches [4–6] and M-matrix theorem [7, 8].

On the other hand, to understand the dynamic behavior of genetic regulatory networks, measurements of all internal states are very useful. The internal states of a genetic regulatory network consist of the concentrations of mRNA and proteins involved in it. However, because of some limitations such as experiment techniques, not all internal states of genetic regulatory network can be effectively measured. As a result, the internal states of genetic regulatory networks cannot be completely available. Therefore, the state estimation problem can play an important role in understanding the dynamic behaviors of genetic regulatory networks. The state estimation problem addressed is to estimate the states based on available output measurements such that the dynamic of estimation error is globally asymptotically stable. Actually, the state estimation methods have been very important in understanding, designing, and controlling dynamic systems such as engineering control system [9], neural networks [10, 11], and complex systems [12].

In this study, we will study the state estimation of genetic regulatory networks with time delays modeled by nonlinear differential equations.  Section 2 briefly describes delayed genetic regulatory networks with SUM regulatory logic. In Section 3 we design a full-order state observer to estimate the states of delayed genetic regulatory networks. Some properties of this observer are discussed. In Section 4, based on LMI approach we establish a sufficient condition under which the dynamic of estimation error for designed state observer is asymptotically and delay-independently stable. In Section 5, a gene repressillatory network is employed to illustrate the effectiveness of our approach described in Section 4.  Section 6 gives our conclusion of this study and points out some directions of future work.

## 2. Delayed Genetic Regulatory Networks

A delayed genetic regulatory network consisting of *n* mRNAs and *n* proteins can be described by the following equations:

(1) m˙i(t)=−kmimi(t)+ci(p(t−τp))p˙i(t)=−kpipi(t)+rimi(t−τm)     for  i=1,2,…,n,

where *m*_*i*_(*t*), *p*_*i*_(*t*) ∈ *R*_+_^*n*^ represent the concentrations of mRNA *i* and protein *i*, respectively. *k*_*mi*_ and *k*_*pi*_ are positive real numbers that represent the degradation rates of mRNA *i* and protein *i*, respectively. *r*_*i*_ is a positive constant representing the rate of translating mRNA *i* to protein *i*. *c*_*i*_(*p*(*t*, *τ*_*p*_)) is a nonlinear function of *p*_1_(*t* − *τ*_*p*_),…, *p*_*n*_(*t* − *τ*_*p*_) representing the regulation function of gene *i*. Both *τ*_*m*_ and *τ*_*p*_ are positive constants indicating time delays of mRNAs and proteins, respectively.

The bottom equation in model (1) describes the translational process. The term *r*_*i*_ *m*_*i*_(*t*) reflects the fact that one kind of proteins is translated only from one kind of mRNA molecules. The top equation in model (1) describes the transcriptional process. One gene or mRNA is generally activated or repressed by multiple proteins in the transcriptional process indicated in the definition of *c*_*i*_(*p*(*t*)). In this paper, we take *c*_*i*_(*p*(*t*)) = ∑_*j*=1_^*n*^ *c*_*ij*_(*p*_*j*_(*t*)), which is called the “SUM” logic [13]. That is, each transcription factor acts additively to regulate gene *i*. The SUM logic is applicable if one gene can be regulated by several proteins independently by binding with different promoters or by a family of similar proteins independently binding to one promoter. In many natural gene networks, this SUM logic does exist [13]. The regulation function *c*_*ij*_(*p*_*j*_(*t*)) is a function of the Hill form [14] as follows:

(2) $$\begin{array}{c} c_{ij}\left(p_{j}\left(t\right)\right)=a_{ij}\frac{1}{1+{\left(p_{j}\left(t\right)/b_{j}\right)}^{h_{j}}} \end{array}$$

if transcription factor *j* is a repressor of gene *i*, or

(3) $$\begin{array}{c} c_{ij}\left(p_{j}\left(t\right)\right)=a_{ij}\frac{{\left(p_{j}\left(t\right)/b_{j}\right)}^{h_{j}}}{1+{\left(p_{j}\left(t\right)/b_{j}\right)}^{h_{j}}} \end{array}$$

if transcription factor *j* is an activator of gene *i*, where *a*_*ij*_ and *b*_*j*_ are nonnegative constants and *h*_*j*_ is the Hill coefficient representing the degree of cooperativity. In this study, assume that *h*_*j*_ ≥ 1. Note that

(4) $$\begin{array}{c} \frac{1}{1+{\left(p_{j}\left(t\right)/b_{j}\right)}^{h_{j}}}=1-\frac{{\left(p_{j}\left(t\right)/b_{j}\right)}^{h_{j}}}{1+{\left(p_{j}\left(t\right)/b_{j}\right)}^{h_{j}}}. \end{array}$$

Then system (1) can be rewritten as follows:

(5) $$\begin{array}{c} \dot{m}\left(t\right)=-K_{m}m\left(t\right)+Gg\left(p\left(t-\tau_{p}\right)\right)+L\\ \dot{p}\left(t\right)=-K_{p}p\left(t\right)+Rm\left(t-\tau_{m}\right), \end{array}$$

where *m*(*t*) = (*m*_1_(*t*),…, *m*_*n*_(*t*)) and *p*(*t*) = (*p*_1_(*t*),…, *p*_*n*_(*t*)); *K*_*m*_ = diag⁡(*k*_*m*_1__,…, *k*_*m*_*n*__), *K*_*p*_ = diag⁡ (*k*_*p*_1__,…, *k*_*p*_*n*__), and *R* = diag⁡ (*r*_1_,…, *r*_*n*_); *G* = (*G*_*ij*_) is an *n* × *n* stoichiometric matrix representing regulatory relationships of the network, which is defined as follows: *G*_*ij*_ = 0 if transcription factor *j* does not directly regulate gene *i*, *G*_*ij*_ = *a*_*ij*_ if transcription factor *j* directly activates gene *i*, and *G*_*ij*_ = −*a*_*ij*_ if transcription factor *j* directly represses gene *i*; *L* = (*l*_1_,…, *l*_*n*_) where *l*_*i*_ is a constant and is defined as *l*_*i*_ = ∑_*j*∈Rep_ *a*_*ij*_, where Rep is the set of repressors of gene *i*. *g* = (*g*_1_,…, *g*_*n*_) where *g*_*j*_(*u*) = (*u*/*b*_*j*_)^*h*_*j*_^/[1 + (*u*/*b*_*j*_)^*h*_*j*_^] is a monotonically increasing function. Obviously these functions with *h*_*j*_ ≥ 1 have the continuous derivatives for *u* ≥ 0. From calculus, we have

(6) $$\begin{array}{c} \theta_{j}=\underset{u\geq 0}{\max}\;g_{j}^{\prime }\left(u\right)=\frac{{\left(h_{j}-1\right)}^{(h_{j}-1)/h_{j}}{\left(h_{j}+1\right)}^{(h_{j}+1)/h_{j}}}{4b_{j}h_{j}}>0. \end{array}$$

## 3. State Observer

In practice, the information about the network states is often incomplete from the experimental measurements. For example, the concentrations of proteins might be immeasurable because of the limitation of measurement techniques. Our purpose of this study is to develop an efficient estimation system (called a state observer) in order to estimate the network states from the available measurements. In this paper, assume that measurements are the linear combinations of mRNA and protein concentrations and thus the output can be expressed as follows:

(7) $$\begin{array}{c} z\left(t\right)=C\left[\begin{array}{c} m\left(t\right)\\ p\left(t\right) \end{array}\right], \end{array}$$

where *z*(*t*) is an *m*-dimensional vector representing the measurements and *C* is an *m* × 2*n* observation matrix. Unless the rank of matrix *C* in (7) is 2*n*, the states of system (5) cannot be exactly estimated from the static observation equation (7) only. In practice, the rank of matrix *C* in (7) is less than 2*n*. To approximately estimate the states of a dynamic system, a dynamic system similar to the original one is designed to estimate the states. In this paper, the full-order state estimator of network (5) is designed as follows:

(8) [m^˙(t)p^˙(t)]=−[Km00Kp][m^(t)p^(t)]+[Gg(p^(t−τp))+LRm^(t−τm)]+D(z(t)−C[m^(t)p^(t)]),

where $\hat{m}(t)$ and $\hat{p}(t)$ are the estimation of states and *D* is 2*n* × *m* estimate gain matrix to be determined.

Let the estimation error be

(9) $$\begin{array}{c} x\left(t\right)=m\left(t\right)-\hat{m}\left(t\right)\;\;y\left(t\right)=p\left(t\right)-\hat{p}\left(t\right). \end{array}$$

Then from (5), (8), and (9), the error system can be described as follows:

(10) $$\begin{array}{c} \left[\begin{array}{c} \dot{x}\left(t\right)\\ \dot{y}\left(t\right) \end{array}\right]=-\left(K+DC\right)\left[\begin{array}{c} x\left(t\right)\\ y\left(t\right) \end{array}\right]+\left[\begin{array}{c} Gf\left(t-\tau_{p}\right)\\ Rx\left(t-\tau_{m}\right) \end{array}\right], \end{array}$$

where *K* = diag⁡(*K*_*m*_, *K*_*p*_) and $f(t)=g(p(t))-g(\hat{p}(t))$.

Now designing the state estimator for network (5) is reduced to find the estimate gain matrix *D* such that the error system (10) is globally asymptotically stable. From (6), we have

(11) $$\begin{array}{c} 0\leq \frac{f_{j}\left(t\right)}{y_{j}\left(t\right)}\leq \theta_{j}\;\;\text{for}\;j=1,2,\ldots ,n. \end{array}$$

Furthermore, for any nonnegative diagonal Λ = diag⁡(*λ*_1_,…, *λ*_*n*_) ≥ 0, from (11) it follows that

(12) −fT(t−τp)2Λf(t−τp)+yT(t−τp)2ΛΘf(t−τp)  ≥0,

where Θ = diag⁡(*θ*_1_,…, *θ*_*n*_).

Once matrix *D* is determined, the estimations $\hat{m}(t)$ and $\hat{p}(t)$ are numerically calculated from (8). That the same technique can be applied for solving (1) directly is the same as solving system (8) with *D* = 0, which results in an estimation error system (10) with *D* = 0. If the system (1) is unstable and the values of $\hat{m}(0)$ and $\hat{p}(0)$ are different from their true counterparts, then the estimation errors will be exponentially increased. Even if the values of $\hat{m}(0)$ and $\hat{p}(0)$ are the exact same as their true counterparts, the round-off errors can also cause the estimation errors to be exponentially increased. Therefore, in practice it is important to design matrix *D* to make sure the estimation error system is stable. Theorems 1 and 2 in next section will guarantee that, for any values of $\hat{m}(0)$ and $\hat{p}(0)$, the estimation errors will be asymptotically converged to zero.

## 4. Main Results and Proofs

In this section we will first derive the conditions under which the error system (10) is globally asymptotically stable for a given estimate gain matrix.

**Theorem 1**

For a given estimate gain matrix *D*, the error system (10) has a unique equilibrium state *x* = 0 and *y* = 0 and is globally asymptotically stable if there exist 2*n* × 2*n* positive definite matrices *P* and *n* × *n* positive definite matrices *Q* and *S* and positive diagonal matrix Λ = diag⁡(*λ*_1_,…, *λ*_*n*_) > 0, such that the following LMI holds:

(13) $$\begin{array}{c} \Omega =\left[\begin{array}{ccc} \Omega_{11} & \Omega_{12} & 0\\ \Omega_{12}^{T} & \Omega_{22} & \Omega_{23}\\ 0 & \Omega_{23}^{T} & -S \end{array}\right]<0, \end{array}$$

where *Ω*_11_ = −(*K* + *DC*)^*T*^ *P* − *P*(*K* + *DC*) + diag⁡(*Q*, *S*), *Ω*_22_ = −diag⁡(2Λ, *Q*), *Ω*_12_ = *P*diag⁡(*G*, *R*), and *Ω*_23_ = [ΛΘ, 0]^*T*^.

**Proof**

Consider the following Lyapunov-Krasovskii functional:

(14) $$\begin{array}{c} V\left(x\left(t\right),y\left(t\right)\right)=V_{1}\left(x\left(t\right),y\left(t\right)\right)+V_{2}\left(x\left(t\right),y\left(t\right)\right), \end{array}$$

where

(15) V1(x(t),y(t))=[xT(t)yT(t)]P[x(t)y(t)],V2(x(t),y(t))=∫t−τmtxT(u)Qx(u)du+∫t−τptyT(u)Sy(u)du.

Differentiating *V*_*i*_(*x*(*t*), *y*(*t*)) defined above along the trajectories of system (10), we have

(16) V˙1(x(t),y(t))=2[xT(t)yT(t)]P[x˙(t)y˙(t)]=−2[xT(t)yT(t)]P(K+DC)[x(t)y(t)] +2[xT(t)yT(t)] ×Pdiag⁡(G,R)[f(t−τp)x(t−τm)],V˙2(x(t),y(t))=xT(t)Qx(t)−xT(t−τm)Qx(t−τm)+yT(t)Sy(t)−yT(t−τp)Sy(t−τp).

Taking inequality (12) into consideration, we have

(17) V˙(x(t),y(t)) ≤V˙1(x(t),y(t))+V˙2(x(t),y(t))  −fT(t−τp)2Λf(t−τp)  +yT(t−τp)2ΛΘf(t−τp) =[xT(t)yT(t)](−2P(K+DC)+diag⁡(Q,S))[x(t)y(t)]  +2[xT(t)yT(t)]Pdiag⁡(G,R)[f(t−τp)x(t−τm)]  −[fT(t−τp)xT(t−τm)]diag⁡(2Λ,Q)[f(t−τp)x(t−τm)]  +2yT(t−τp)[ΛΘ0][f(t−τp)x(t−τm)]  −yT(t−τp)Sy(t−τp) =ξT(t)Ωξ(t)<0,

where *ξ*(*t*) = [(*x* ^*T*^(*t*), *y* ^*T*^(*t*)), (*f* ^*T*^(*t* − *τ*_*p*_), *x* ^*T*^(*t* − *τ*_*p*_)), *y* ^*T*^(*t* − *τ*_*p*_)]^*T*^.

From Lyapunov-Krasovskii theory [15], the error system (10) is globally asymptotically stable. From (10), *x* = 0 and *y* = 0 are an equilibrium state. To prove the uniqueness of the equilibrium state of the error system (10), here we use proof-by-contradiction technique. Note that Lyapunov-Krasovskii functional (18) associated with the error system (10) is independent of the equilibrium state. Therefore if the error system (10) has another equilibrium state, it is also globally asymptotically stable, which is not possible.

In Theorem 1, for a given estimate gain matrix *D*, the stability condition of the error dynamic system (10) is established in terms of linear matrix inequality (LMI) which can be solved by standard MATLAB function. If matrix *D* is unknown, matrix inequality (13) becomes nonlinear in matrices, *P*, *D*, *Q*, *S*, and Λ, which is not easy to be solved. However, let *PD* = −*T*; then matrix inequality (13) becomes linear in matrices, *P*, *T*, *Q*, *S*, and Λ. Therefore, we have the following theorem.

**Theorem 2**

If there exist 2*n* × 2*n* positive definite matrices *P* and *n* × *n* positive definite matrices *Q* and *S*, positive diagonal matrix Λ = diag⁡(*λ*_1_,…, *λ*_*n*_) > 0, and an 2*n* × *m* matrix *T* such that the following LMI

(18) $$\begin{array}{c} \Omega =\left[\begin{array}{ccc} \Omega_{11} & \Omega_{12} & 0\\ \Omega_{12}^{T} & \Omega_{22} & \Omega_{23}\\ 0 & \Omega_{23}^{T} & -S \end{array}\right]<0 \end{array}$$

holds, where *Ω*_11_ = −*KP* − *PK* + *C* ^*T*^ *T* ^*T*^ + *TC* + diag⁡(*Q*, *S*), and sub-matrices *Ω*_22_, *Ω*_12_, and *Ω*_23_ are the same as in Theorem 1, then with the estimator gain matrix

(19) $$\begin{array}{c} D=-P^{-1}T \end{array}$$

the error system (10) has a unique equilibrium state *x* = 0 and *y* = 0 and is globally asymptotically stable.

Proof of Theorem 2 is straightforward from Theorem 1 and thus is omitted here.

## 5. An Illustration Example

**Figure 1:**
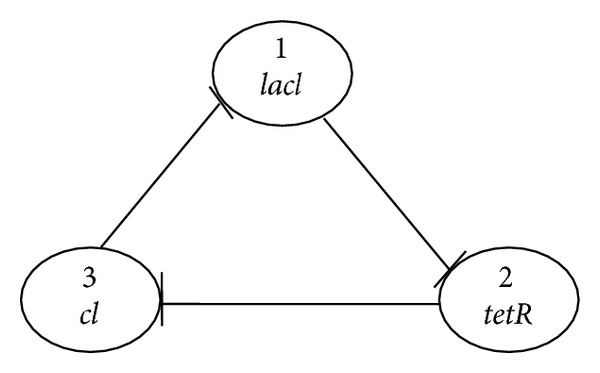
Structure of gene repressilatory network.

In this section, we employ the gene repressilatory network to show the effectiveness and correctness of our theoretical results. The gene repressilatory network consists of three genes and three proteins (*lacl*,* tetR*, and* cl*), each repressing the transcription of its downstream partner [16] as shown in Figure 1. This network without time delays has been studied theoretically and experimentally in [16]. The delay-independent local and global stability of this gene repressilatory network with time delays has widely been studied in [1–8].

The mathematical model of this gene repressilatory network with time delay is described by the following equation:

(20) $$\begin{array}{c} {\dot{m}}_{i}\left(t\right)=-k_{m}m_{i}\left(t\right)+\frac{a}{1+p_{i-1}^{h}\left(t-\tau_{p}\right)},\\ {\dot{p}}_{i}\left(t\right)=-k_{p}p_{i}\left(t\right)+rm_{i}\left(t-\tau_{m}\right), \end{array}$$

where *k*_*m*_, *a*, *k*_*p*_, and *r* are positive constants and subscript 0 = 3.

In this study we consider gene repressillatory network (20) with the values of parameters set as follows: *h* = 2, *k*_*m*_ = 1.2, *a* = 2.5, *k*_*p*_ = 1, and *r* = 0.8. For system (20) with these parameter specifications, we have

(21) $$\begin{array}{c} K_{m}=\left[\begin{array}{ccc} 1.2 & 0 & 0\\ 0 & 1.2 & 0\\ 0 & 0 & 1.2 \end{array}\right],\;\;G=\left[\begin{array}{ccc} 0 & -2.5 & 0\\ 0 & 0 & -2.5\\ -2.5 & 0 & 0 \end{array}\right],\\ K_{p}=\left[\begin{array}{ccc} 1 & 0 & 0\\ 0 & 1 & 0\\ 0 & 0 & 1 \end{array}\right],\;\;R=\left[\begin{array}{ccc} 0.8 & 0 & 0\\ 0 & 0.8 & 0\\ 0 & 0 & 0.8 \end{array}\right]. \end{array}$$

And $\theta_{j}=3\sqrt{3}/8$ for *j* = 1,2, 3.

**Case A**

Assume that the concentration of all proteins is unable to be measured. The observation matrix *C* is

(22) $$\begin{array}{c} C=\left[\begin{array}{cccccc} 1 & 0 & 0 & 0 & 0 & 0\\ 0 & 1 & 0 & 0 & 0 & 0\\ 0 & 0 & 1 & 0 & 0 & 0 \end{array}\right]. \end{array}$$

By using MATLAB LMI toolbox, we solve LMIs (18) with the above data for *P*, *T*, *Q*, *S*, and Λ and obtain

(23) $$\begin{array}{c} P=\left[\begin{array}{cccccc} 1.8750 & 0 & 0 & 0 & 0 & 0\\ 0 & 1.8750 & 0 & 0 & 0 & 0\\ 0 & 0 & 1.8750 & 0 & 0 & 0\\ 0 & 0 & 0 & 8.3807 & 0 & 0\\ 0 & 0 & 0 & 0 & 8.3807 & 0\\ 0 & 0 & 0 & 0 & 0 & 8.3807 \end{array}\right],\\ T=-\left[\begin{array}{ccc} 10.1248 & 0 & 0\\ 0 & 10.1248 & 0\\ 0 & 0 & 10.1248\\ 0 & 0 & 0\\ 0 & 0 & 0\\ 0 & 0 & 0 \end{array}\right],\\ Q=\left[\begin{array}{ccc} 9.2810 & 0 & 0\\ 0 & 9.2810 & 0\\ 0 & 0 & 9.2810 \end{array}\right],\\ S=\left[\begin{array}{ccc} 7.9497 & 0 & 0\\ 0 & 7.9497 & 0\\ 0 & 0 & 7.9497 \end{array}\right],\\ \Lambda =\left[\begin{array}{ccc} 6.9963 & 0 & 0\\ 0 & 6.9963 & 0\\ 0 & 0 & 6.9963 \end{array}\right]. \end{array}$$

Therefore, we have

(24) $$\begin{array}{c} D=-P^{-1}T=\left[\begin{array}{ccc} 5.4 & 0 & 0\\ 0 & 5.4 & 0\\ 0 & 0 & 5.4\\ 0 & 0 & 0\\ 0 & 0 & 0\\ 0 & 0 & 0 \end{array}\right]. \end{array}$$

**Figure 2:**
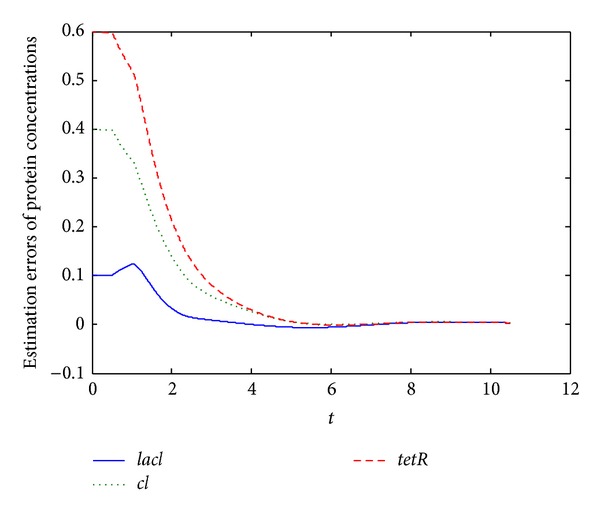
Estimation errors of protein concentrations of system (20) with specified parameters and *τ*_*p*_ = *τ*_*m*_ = 0.5 minutes while mRNA concentrations are available.

Figure 2 depicts the estimation errors of protein concentrations of delayed genetic regulatory network (20) with specified parameters in the caption of Figure 2. From Figure 2, it can be seen that in six minutes the estimated protein concentrations are exactly the same as the true protein concentrations although they are not measured. The time that needs to exactly estimate the true states depends on the initial errors between the true states and estimated states (which are random guesses in practice). In Figure 2, the initial errors of protein estimations range from 0.1 to 0.6. If the initial errors are zero, the estimated state would be the exact true states from beginning on.

**Case B**

Assume that the concentration of all proteins is able to be measured while we would like to estimate the gene expressions. The observation matrix *C* becomes

(25) $$\begin{array}{c} C=\left[\begin{array}{cccccc} 0 & 0 & 0 & 1 & 0 & 0\\ 0 & 0 & 0 & 0 & 1 & 0\\ 0 & 0 & 0 & 0 & 0 & 1 \end{array}\right]. \end{array}$$

By using MATLAB LMI toolbox, we solve LMIs (18) with above data for *P*, *T*, *Q*, *S*, and Λ and obtain

(26) $$\begin{array}{c} P=\left[\begin{array}{cccccc} 19.3747 & 0 & 0 & 0 & 0 & 0\\ 0 & 19.3747 & 0 & 0 & 0 & 0\\ 0 & 0 & 19.3747 & 0 & 0 & 0\\ 0 & 0 & 0 & 21.9879 & 0 & 0\\ 0 & 0 & 0 & 0 & 21.9879 & 0\\ 0 & 0 & 0 & 0 & 0 & 21.9879 \end{array}\right],\\ T=-\left[\begin{array}{ccc} 0 & 0 & 0\\ 0 & 0 & 0\\ 0 & 0 & 0\\ 53.1451 & 0 & 0\\ 0 & 53.1451 & 0\\ 0 & 0 & 53.1451 \end{array}\right],\\ Q=\left[\begin{array}{ccc} 17.6592 & 0 & 0\\ 0 & 17.6592 & 0\\ 0 & 0 & 17.6592 \end{array}\right],\\ S=\left[\begin{array}{ccc} 75.9017 & 0 & 0\\ 0 & 75.9017 & 0\\ 0 & 0 & 75.9017 \end{array}\right],\\ \Lambda =\left[\begin{array}{ccc} 69.2189 & 0 & 0\\ 0 & 69.2189 & 0\\ 0 & 0 & 69.2189 \end{array}\right]. \end{array}$$

Therefore, we have

(27) $$\begin{array}{c} D=-P^{-1}T=\left[\begin{array}{ccc} 0 & 0 & 0\\ 0 & 0 & 0\\ 0 & 0 & 0\\ 2.4170 & 0 & 0\\ 0 & 2.4170 & 0\\ 0 & 0 & 2.4170 \end{array}\right]. \end{array}$$

**Figure 3:**
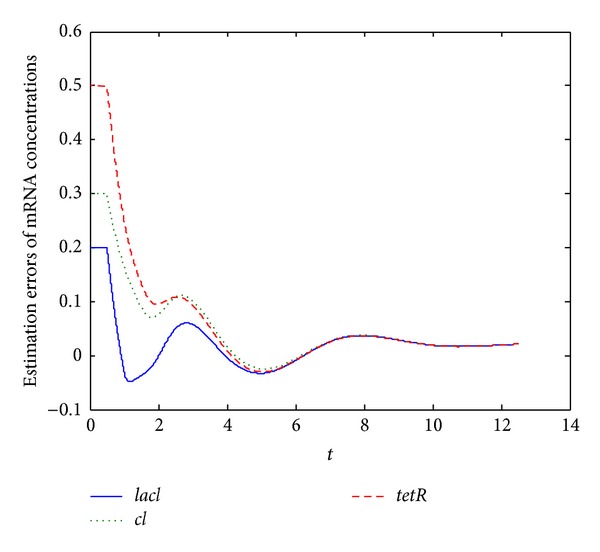
Estimation errors of mRNA concentrations of system (20) with specified parameters and *τ*_*p*_ = *τ*_*m*_ = 0.5 minutes while protein concentrations are available.

Figure 3 depicts the estimation errors of mRNA concentrations of delayed genetic regulatory network (20) with specified parameters in the caption of Figure 3 and knowing protein concentrations. From Figure 3, it can be seen that in about ten minutes the estimated mRNA concentrations can pretty well approximate the true mRNA concentrations although they are not measured. In Figure 3, the initial errors of protein estimations range from 0.2 to 0.5.

## 6. Conclusion and Future Work

In this paper, we have studied the state estimation of genetic regulatory networks with time delays. Based on LMI approach, a full-order state observer is designed to estimate the states from incomplete measurements so that the state estimation error is globally asymptotically stable. The theorems presented in this paper have been illustrated by the gene repressillatory network. The simulation results have verified that our designed observer can effectively estimate the unmeasured states. In this study, we assume that all parameters of genetic regulatory networks are available. In practice, some of parameters in networks may be unknown. One direction of our future work is to employ the extended Kalman filter [17] to estimate the known parameters and state of the systems simultaneously. Parameter uncertainties and noise perturbations exist in genetic regulatory networks [4, 6, 7, 16, 18] and measured outputs, which can affect the performance of state observer. The second direction of our future work is to design robust state observer for genetic regulatory networks with parameter uncertainties and noises. Typically measured outputs are sampled at a series of time points although state variables of genetic regulatory networks are continuous. The third direction of our future work is to design a state observer for genetic regulatory networks with discretized outputs.
